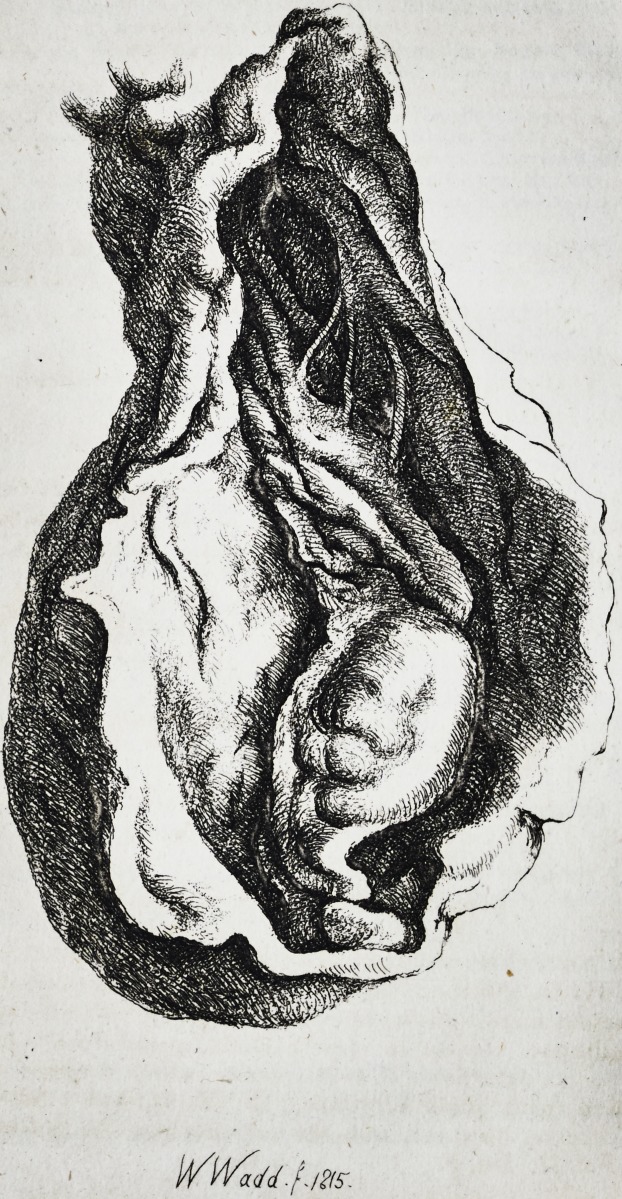# Essays on Hydrocephalus Acutus; or Water in the Brain

**Published:** 1820-04-01

**Authors:** 


### Medico-Chirurgical Journal;

OR,

LONDON MEDICAL AND SURGICAL

REVIEW.

Nec tibi quid liceat sed quid fecisse decebit
Occurrat mentemque domat respectus honesti. CLAUD.

No. 8.] APRIL 1, 1820. [VOL. II.

### I.

*I. Essays on Hydrocephalus Acutus; or Water in the Brain.* By J. CHEYNE, M.D. F.R.S.E. &c. One Vol. Octavo, Second Edition, 168 pages. London, 1819.

*II. Hydrocéphale Argüe essentielle, et Symptomatique.* Par M. VAIDY. *Dict. des Sciences Medicales.*

*III. Memoire sur L'Hydrencéphale, ou Céphalite interne Hydrencéphalique.* Par J. F. COINDET, M.D. Medecin en Chef des Hospices de Genève, &c. Octavo, pp. 292. Genève, 1818.

*IV. A Statement of the Early Symptoms which lead to the Disease termed Water in the Brain, &c. with an* APPENDIX to ditto. By G. D. YEATS, M. D. F. R. S. Fellow of the Royal College of Physicians, London. 1817 and 1819.

*V. Of Apoplexia Hydrocephalica, or Hydrocephalus Internus.* By JOHN COOK, M. D. F. A. S. Fellow of the Royal College of Physicians, &c. &c.

[Treatise on Nervous Diseases, Chap. viii.]

THE ancient physicians had but a very imperfect notion of Hydrocephalus. Hippocrates does not mention it, and Areteus merely enumerates it among the different species of dropsy. Galen is more particular, and divides hydrocephalus externus into four species, which division is followed, with some additions, by Ætius, and others. It is curious, however, that the ancients describe *hydrocepha-*

*lus externus* as very common in their time, whereas it is now an extremely rare occurrence. Could this be owing to the violence offered to the heads of infants by the rude and unskilful midwifes of old? Rhazes is the only one of the Arabians who hints at hydrocephalus *internus* when speaking of a morbid enlargement of the head, from an accumulation of aqueous fluid in the interior of the cranium. It is only among the moderns, who have been aided in their researches by the light of morbid anatomy, that we are to look for any precise and certain knowledge respecting this important disease.

We shall divide hydrocephalus acutus into two species, the idiopathic and the symptomatic.

1. *Idiopathic Species.* The definition of this species might be briefly conveyed in the following terms. "*A rather sudden effusion of serous fluid into the ventricles, or on the surface of the brain, from the tunica arachnoides or pia mater, idiopathically affected.*"

Our own countrymen led the way in the investigation of this disease. Yet, about ten years before Whytt and Fothergill published their opinions and observations on dropsy of the ventricles, Meyserey, in a work on the diseases of soldiers, gives no bad description of the complaint, under the name of *brain fever*. In the writings of Pringle and Huxham also, may be found scattered hints of a similar tendency; but in all these, the effusion was considered as a termination, accompaniment, or consequence of some other disease. Cullen, for instance, denominates it a variety of apoplexy, "Apoplexia Hydrocephalica," and Pinel, in the *first* edition of his *Nosographie Philosophique*, considers hydrocephalus acutus as a variety of *brain fever*, in imitation of Chardel. But in the *last* edition of his work, this author restores the disease to its proper rank, as an idiopathic affection, and places it in the class of Dropsies.

*Causes of Hydrencephalus Acutus.* Let it be remembered, that we are here speaking of the *idiopathic species*, which will account for the limited range of occasional and exciting causes. Youth appears to be what we may term the predisposing period; particularly that which intervenes between the two dentitions. Peculiarities of *hereditary organization* are now well known causes, especially the strumous diathesis, characterized by a delicate, irritable, and often beautiful frame of body, with a corresponding acuteness of intellect, and liveliness of disposition.

Among the more tangible *exciting* causes of the complaint, we may reckon injuries or commotions of the head by blows or falls. We have seen several instances of this kind, and we suspect that it is more frequently the case than is believed. Violent mental emotions, by throwing the heart into inordinate action, and thus pouring extraordinary currents of blood on the delicately organized brain, conduce to the developement of the disease in the predisposed. The neglect or maltreatment of children among the poor, where they are left crying, almost to convulsions, for hours together, must lead, in many instances, to effusion in the brain. The suppression of a spontaneous or habitual evacuation from the system, as of nasal hæmorrhage, diarrhœa, or eruptions about the head in dentition, form a fruitful source of irritation on the coverings of the brain, and consequent effusion. And the period of dentition itself is very often an exciting cause, especially if the inflammatory condition of the child, at that time, be not properly attended to.

There is little doubt also, that particular constitutions of the air, which determine other epidemic diseases, as scarlatina, measles, hooping cough, &c. produce a tendency to hydrocephalic irritation. Why should not the membranes and vessels of the brain be under the influence of atmospheric causes, as well as those of the lungs, intestines, &c.? Itard, a French physician of eminence, distinctly asserts, that during two years when hydrocephalus was peculiarly prevalent, the one was remarkable for an epidemic scarlatina, the other for an ataxique fever among children.* Vieusseux observed this disease epidemic at Geneva.

*Proximate Cause.* Dr. Quin thought he had made a notable discovery in separating hydrocephalus acutus from *dropsy*, with which Whytt, Fothergill, and others had confounded it, and places it to the account of a morbid circulation or accumulation of blood in the vessels of the brain, sometimes rising into a state of inflammation, and terminating in effusion. Dr. Cheyne adopts a nearly similar theory, conceiving "that in this disease there is produced a venous congestion, in addition to, and probably arising from, *the increased arterial action*:—that the effusion of serous fluid arises from this venous congestion:—that this effusion has a tendency to counteract the baleful effects of the increased action, and to retard the fatal termination of the disease: of course, that the effusion into

* Dict. des Sciences Med. Vol xxii. p. 223.

the ventricles is not the cause of the violent symptoms; and, that the increased arterial action, though perhaps varied, does not cease when the congestion and effusion have taken place."

The late Dr. Rush, of America, was one of the first who began to have more rational ideas respecting the nature and treatment of this disease, by directing the attention of practitioners to the vascular activity of the vessels of the brain, and to the advantages of blood-letting; and by the following remark, approached nearer to the facts than any doctrine which had been hitherto published. "No more," he says, "occurs in this disease than what takes place where hydrothorax follows inflammation of the lungs, or where serous effusions follow inflammation of the joints." *Inquiries, Vol ii. p.* 216.

Now, the researches *post mortem*, and the most successful modes of treatment, of modern physicians, have proved, almost to a demonstration, that the above observations will strictly apply to *to all acute dropsies*, wherever situated; whether in the bags of the pleura, or among the convolutions of the intestines. The proximate cause, therefore, of hydrocephalus acutus, is the same as that of acute hydrothorax or ascites. As far as we can judge by symptoms and by dissections, we have every reason to believe that an IRRITATION, more or less approaching to INFLAMMATION, is determined on the arachnoid and pia matral tissues of the brain, producing, if not checked, a superabundant serous exhalation from their surfaces, in the same way as the inflammatory irritation of acute rheumatism, when determined on the synovial tissue of the knee-joint will, in a short time, fill the articular cavity with a serous exudation. How far a diminished action of the absorbents may be concerned in this state, we cannot tell; but we know, with certainty, that an irritated or inflamed membrane will throw out a preternatural quantity of fluid. We entirely agree, therefore, in the following sentiments of the elder Dr. Parry, whose Elements of Pathology, we are sorry to see so little read or attended to by the profession, *in general.*

"Whether, says this excellent physician, inflammation exist in acute hydrocephalus, or not, all the previous and concomitant symptoms are those which shew increased impetus; and thus, if the general principle which I have endeavoured to establish with regard to dropsies, be well founded, this disease, and the other symptoms attributed to increased determination of blood to the brain, throw mutual light on each other." *Elements of Pathology, &c.* 352.

*Symptomatology.* In no disease is there a greater difficulty of laying down a general description of symptoms than in Hydrocephalus, so proteiform are these symptoms themselves. We can only delineate the more prominent and constant features. In *idiopathic* hydrocephalus there is, for the most part, pain in the head, more or less acute, from the beginning, accompanied by a preternatural irritability in the organs or nerves of sense, especially of the eye and ear. Light annoys the child, and sounds which, in health, would produce little or no sensation, now cause him to start, or even cry out. When in bed, the child is constantly rolling about its head on the pillow, or putting its hands up towards its face, while slight agitation may occasionally be observed over the whole frame. A febrile state, especially towards the evening, may now be detected. The pain in the head frequently alternates with pain in the back of the neck, in the shoulders, arms, or pectoral muscles. The expression of countenance is perpetually changing, as is the rythm of the pulse, and the temperature of the body. The tongue is generally clean, or only slightly furred; the epigastrium tender, particularly when the head ache is least complained of. There is marked depression of strength, except during the presence of convulsions; the functions of the digestive organs are impaired; the evacuations are yellow or clayey, slimy, and fœtid, and they speedily turn green on exposure to the air. The urine is high coloured or turbid, and the sediment is of a white, glairy nature. The stomach is generally though not always irritable. The eye is much looked to in hydrocephalus; but, like the pulse in other diseases, it is very fallacious. In the stage of irritation it is morbidly sensible, and the pupil closely contracted; but as effusion takes place, the pupil widens, and the eye loses its lustre and expression, till at length strabismus itself ensues with convulsions of the whole body.

The stage of irritation or excitement is of various duration, from two or three days to as many weeks; and this slow or quick progress has led to the very useless division of the disease into different forms, according to the march or violence of the symptoms.

When effusion has once taken place, although the symptoms of inflammation and irritation experience a considerable mitigation, they do not entirely subside; and this circumstance renders it very difficult to ascertain the exact period of effusion, and consequently the time for changing our mode of treatment. When the fluid is extravasated to a certain extent, the symptoms of a compressed brain, as stupor, grinding of the teeth, convulsions, hemiplegia, projection of the eyes, squinting, blindness, injection of the conjunctiva, puffiness of the face, and, finally, apoplexy ensue, and terminate the scene.

Nothing, however, can be more irregular, as was before observed, than the progress and development of the foregoing symptoms. Very often the head-ache is not complained of till effusion has actually taken place. At other times, the most alarming symptoms will suddenly remit; and during this remission, the little patient will appear in almost its ordinary health, and that too when it is on the very verge of the fatal goal! The child's sensibility returns; the fever disappears; he recovers his usual liveliness; returns to his play; takes nourishment; and all danger seems to vanish. The parents, and even the medical attendant, deliver themselves up to hope and joy; but, all at once, the scene changes; the symptoms of effusion on the brain return with terrible rapidity, and convulsions quickly terminate the life of the little patient.

These flattering remissions of the symptoms of hydrocephalus may sometimes be accounted for by evident changes in the balance of the circulation at the time, or by an increased determination to, or discharge from, other parts of the system. Thus they have been seen to subside on the breaking out of a profuse salivation from mercury; on the establishment of a great discharge from blistered surfaces, or the sudden supervention of œdema of the lower extremities. These, of course, relieve for a time the cerebral congestion, on which the symptoms depend, but do not always secure the organ from a subsequent attack.

In this disease, as in apoplexy, it is not absolutely necessary that there should be effusion from the vessels before death ensues. An epidemic hydrocephalus prevailed at Geneva in the year 1805, and terminated fatally from the second to the fourth day, without any very alarming symptoms. Frequently, on dissection, no water was effused, and death seemed to result, in such cases, from the violence of the cerebral irritation, or perhaps engorgement of the cerebral vessels. Upon the whole, there is the greatest uncertainty in every thing that respects hydrocephalus, except its two principal stages; the *first*, of cerebral irritation, the *second* of cerebral compression. The prominent characters of the *former* are head-ache, gastric irritability, agitation, delirium, fever more or less developed, morbid sensibility of the retina, pain or tension of the epigastrium, and of the cervical region. Those of the *latter* are dilatation of the pupil, strabismus, drowsiness or stupor, œdema of the face, paralysis, convulsions, insensibility.

Dr. Cook, after stating the various opinions of authors, relative to the diagnosis of hydrocephalus, concludes thus:

"From what I have read and seen, I am of opinion that when, in addition to fever, with vomiting and other symptoms of deranged stomach and bowels, we observe marks of uneasiness or pain in the head, drowsiness, morbid sensibility to light, a disposition to the horizontal posture, the face being turned from the light, and the hands placed round the head, there is strong reason to believe that water is actually effused in the brain. When the pulse, from being frequent and regular, becomes slow and irregular, when violent pain in the head, with screaming, or a comatose state supervenes, when the pupil of the eye becomes either preternaturally dilated or contracted, with strabismus, the disease is still more manifest; but when the pulse, from having been slow, has become again very frequent, when the fever is very high, with flushings in the face, and inflamed eyes, when delirium in a great degree, or perfect stupor, with other symptoms, mentioned towards the end of Dr. Whytt's description, are present, I think there can be no doubt either of the nature or degree of the complaint." 433.

### II. *Second Species, or Symptomatic Hydrocephalus.*

Such are the prominent traits of *idiopathic* hydrocephalus, a disease which undoubtedly exists, though it is much less common than the species which we are now about to describe. If it be less common, however, it is more fatal than the symptomatic species, and it is probably by confounding the two together, that we have so many discrepancies of opinion respecting the curability or incurability of the disease. Whytt, Fothergill, Watson, and some others, have laid down a most unfavourable prognosis; while Lettsom, Willan, Percival, Cheyne, Yeats, and many others, represent the disease, in its first stage, as greatly under the control of human art.

The *symptomatic* hydrocephalus occurs, perhaps, fifty times, where the idiopathic species occurs once; and it is from that species the great majority of our best descriptions of the disease are drawn, especially those of the *premonitory* symptoms. It cannot be expected, indeed, that an idiopathic cerebral irritation can long exist, without some degree of derangement in *all* the functions of the body, more especially those most commonly associated with the brain, as of the digestive organs; but if the case be carefully observed, it will be found that these are consecutive of the cerebral affection, and that they are, in general, slighter in degree than where they precede or give origin to the hydrocephalic irritation. It is the same with the hydrocephalic irritation itself; it is always more manageable when symptomatic than when idiopathic.

In childhood the digestive organs and the brain have a greater supply of blood, and a higher degree of excitability, than any other parts in the body. The *former*, till puberty, have an excessive function to perform, in building up the corporeal fabric; the brain, as the centre of the nervous system, to which all impressions are transmitted, is at that period endued with the most vivid sensibility. These two systems, then, are liable to numerous derangements, from the morbid stimulation of food and drink on the one, and moral irritation on the other. The two systems also act and react upon each other perpetually, and one cannot long continue disordered without drawing the other into a similar state.

A morbid state of the digestive organs so very *generally* propagates an irritation to the brain, that some intelligent physicians have been led into the opinion that hydrocephalus is *always* a secondary, or consecutive link in the chain of disease. Derangement of function or structure in the livers of children will, it is well known, very often induce all the symptoms, and all the fatal consequences of hydrocephalus. This was long ago insisted on by Professor Heineken of Bremen, and more lately by Doctors Cheyne and Curry; the latter, pushing the theory to extravagance, conceived that *all* cases of hydrocephalus were determined by a previous erythema or irritation of the liver. This circumstance only goes to prove, however, that such is OFTEN the case. More recently, Dr. Yeats of London, Dr. Wilson of Kelso, and Dr. Ayre of Hull, have brought forward powerful evidence of hydrocephalus being a consequence of disordered states of the chylopoietic viscera.

The following are Dr. Cheyne's reasons for considering hydrocephalus as very frequently dependent on abdominal derangements, as stated in his recent publication.

"1. Cases must have occurred to every physician, of children especially in the lower ranks of life, relieved by a short course of active purgative medicines, from a situation in which much of the expression, and many of the symptoms of hydrocephalus were combined — irregular fever, retching, head-ache, lethargy to a great extent; symptoms which evidently arise from a disordered state of the abdominal viscera.

"2. In many cases, previously to the appearances of hydrocephalus, the chylopoietic viscera have been disordered for many weeks.

The appetite has been impaired, the bowels costive, the stools betraying disorder in the hepatic system; there has been all that want of alacrity, both of body and mind, so invariably the consequence of derangement of the biliary secretion; and in several children, previous to the existence of any morbid sensation, the first symptom of indisposition has been the loss of the healthy colour of the skin.

3. In children predisposed to hydrocephalus, while removing a vitiated biliary secretion and disordered state of the bowels, by a course of purgative medicines, in which mercury was generally an ingredient, the very same symptoms have been removed which had presented themselves in other children of the same family, when attacks of hydrocephalus, which actually proved fatal, were supposed to be established.

4. In children who had not any known tendency to disease, the symptoms, which are always found in the beginning of hydrocephalus, have often been removed by the same means.

5. In many cases it has been remarked, that children in the early stage of hydrocephalus, when the region of the liver was pressed upon, have complained much more than they did when the same pressure was applied to any other part of the abdomen.

6. In dissections after hydrocephalus, I have found the liver inflamed, adhering preternaturally to the peritoneum, enlarged, studded with tubercles, and otherwise differing from its sound state.

7. In one or two cases, alarming from their great resemblance to hydrocephalus, during recovery, the enlarged liver has been greatly reduced by mercurial purgatives.

In what way this morbid sympathetic influence is propagated we cannot, in the present state of our knowledge, say with certainty; but the *fact* is not the less true on that account. We may pretty safely aver, that it is through the nervous or vascular system, or both. Every man's experience has demonstrated the frequency of headache from irritation in the *primæ viæ*, especially the stomach. This must be principally through the medium of the nerves. Now a strong degree of, or often repeated irritation, will, in a predisposed organ or part, soon induce inflammation; and this appears to be the case where hydrocephalus is determined by a morbid state of the digestive organs. A congested state of the liver, or a loaded condition of the intestines, may very easily be conceived to derange the balance of the circulation, and throw an undue proportion of the vital fluid on the brain. If a morbid *irritation* have been previously propagated to the encephalon from the digestive viscera, this increased afflux of blood will considerably aggravate the cerebral irritability; for it is as true that, *ubi fluxus ibi irritabilitas,* as the well known fact that *ubi irritatio, ibi fluxus.* Dr. Yeats has lately given some most interesting views of this subject, in the Appendix to his preceding work, and of which some account was presented in a former number of this Journal. Dr. Cheyne has also enlarged on this topic, in his recent valuable publication, to which we refer the reader.

*Hydrocephalus Symptomaticus* creeps on in an insidious manner; but an attentive observer will perceive the predominance of the *abdominal* derangements in the premonitory and early stages of the disease, and will often prevent the hydrocephalic symptoms entirely by removing the primary source of irritation.

In this form of the disease, the following, among many other premonitory symptoms, will commonly prevail; viz. languor, as if from fatigue; an unhealthy aspect of the countenance, evinced by transient paleness and collapse of the features; dark-coloured line under the eye, and dullness of that organ; the skin loses its soft pliable feel, and becomes harsh, with an increase of temperature. The appetite becomes capricious, with occasional thirst; the bowels torpid or irregular; the tongue white; the urine high-coloured, and sometimes sedimentous. All this time the vascular system remains tranquil, or but little affected. When the bowels are moved, unnatural secretions will, almost always, be perceived; the fæces will be sometimes too light in colour; sometimes greenish, clayey, or yeasty, with unpleasant fœtor.

"Stools of the same nature with those which are generally passed in hydrocephalus, are seldom seen in any other disease. They resemble boiled laver, forming a dark green gelatinous mass, with an oily-looking surface, and are of a sickly but not fœtid smell; they consist of flakes of inspissated bile, which gives them their colour, and of the mucus of the intestines. Diffused in water, they do not render it turbid, and scarcely change its colour; it seems as if the vitiated bile, by irritating the surface of the intestines, occasioned the copious discharge of mucus to which the stools owe their consistence. When such stools once appear, they in general continue, till the disease is terminated by the death of the patient; and though I have known them made more copious, yet their character is seldom changed by a perseverance in the use of drastic purgatives. Unless the irritation in the bowels is allayed, the biliary secretion increased, and its nature changed, our efforts will be unavailing. Common cathartics increase the irritation of the bowels, which it is one of our great objects to diminish, and also carry the mercury out of the system before it has had time to make a sufficient impression upon it." Cheyne, P. 64.

If the abdomen be examined at this time, a puffiness or fullness of the epigastric region will be perceptible, with tenderness on pressure; the sleep is disturbed, and there will be some degree of emaciation, from the defective function of nutrition. All this time, there will be little more than uneasiness, rather than pain of the head, with some sense of external soreness. Children at this period, as Dr. Yeats has remarked, will often evince a precision of ideas and quickness of apprehension much beyond their years, and thus become interesting objects to their friends, rendering their loss more sensibly felt afterwards.

Upon this premonitory state of chylopoietic derangement, the symptoms of cerebral irritation more or less rapidly supervene, and then the train of phenomena characteristic of this accessory and dangerous affection, evince themselves in the manner already described under the head of *idiopathic hydrocephalus*, from which the symptomatic species cannot, in fact, be distinguished, after the disease is formed. Dr. Ayre, who has described this premonitory state of derangement in the digestive organs, under the name of marasmus, justly observes, that there is, in fact, often considerable difficulty, especially in infancy, to determine where the symptoms proper to the marasmus terminate, and those belonging to hydrocephalus internus begin.

But it is not derangement of the abdominal viscera alone that leads to cerebral irritation and effusion. Any febrile commotion in the system of a child, predisposed constitutionally to this insidious disease, will endanger the developement of the complaint; for instance, the common remittent fever of infants, scarlatina, or any of the eruptive fevers. In such cases, the brain being the weakest organ, or that most disposed to irritation and inflammation, it suffers during the general excitement of the system, and hydrocephalus is the result.

The irritation of dentition is peculiarly liable to be propagated to the membranes of the brain, and there not only *simulate*, but produce the genuine symptoms of hydrocephalus. We say *simulate;* for we believe that, in many cases, the hydrocephalic phenomena are only *simulated* in dentition, because we have seen them all removed by the lancing of a gum, and a little aperient medicine, which would hardly have been the case, had the disease been actual hydrocephalus. In this way, many cures have been said to be effected, and remedies unmeritedly extolled, where the disease was counterfeited. Nevertheless, if this *simulated* affection be allowed to continue unchecked, in such young and irritable constitutions, it may, and no doubt often does, terminate in real hydrocephalus.

Post mortem Researches. It ought to be borne in mind that *irritation*, and even the primary stages of *inflammation*, are only lesions of the *vital properties* of parts; and that if these lesions occasion death [which, in such an organ as the brain, they frequently do] before they have altered the *structure*, or given rise to morbid *secretions*, they will *not* be cognizable on dissection. We are not therefore to conclude, that a disease was not hydrocephalus, because, on dissection, no effusion appears in the ventricles, or at the base of the brain. If, therefore, a child be suddenly cut off, while labouring under the usual symptoms of hydrocephalus, and dissection shews no adequate cause of death, we may fairly presume that the fatal event took place from *lesion of the vital properties* of the brain, and before the structure was altered, or any water effused. The child probably dies in consequence of the vital energy of the brain being destroyed, either by too great irritation, or morbid vascular activity. The late Dr. Kirkland, of Ashby-de-la-Zouch, has some very good remarks on this subject, in his communications on apoplectic and paralytic affections.

In the majority of cases, however, both the causes and effects of the disease are sufficiently demonstrable in the brain after death. The principal, and the most common phenomena which present themselves on removing the skull-cap, are, a gorged state of the sinuses of the dura mater, and of the vessels spread over the surface of the brain. It is somewhat curious, that the vessels are sometimes distended with an æriform fluid,[1] as was observed by Morgagni, Lieutaud, and Portal, in examining apoplectic bodies. The medullary mass itself, though sometimes soft and flabby, is, for the most part, rather firmer than natural.

Dr. Porter, of Bristol, in a most valuable paper on this subject, published in the third number of the Medico-Chirurgical Journal, has endeavoured to show, that genuine hydrocephalus acutus depends on a subacute inflammation of the *posterior* arteries of the encephalon, and that the symptoms during life distinguish, in many cases, this state from meningeal inflammation constituting true phrenitis. This ingenious and able physician, however acknowledges that the two diseases "often glide imperceptibly into each other, as the inflammatory condition of the encephalon becomes more general." His conclusions are strongly supported by the dissections which he has made. In these dissections, was seen an abundance of gelatinous matter, and of coagulable lymph, covering the pons varolii and adjacent parts—unequivocal products of an inflammatory condition. In fourteen brains of patients dying of this disease, he did not, in one instance, find this appearance wanting.*

Dr. Cheyne remarks—"upon dissection, we generally find within the cranium, the veins, particularly those of the membranes on the surface of the brain, and lining of the ventricles, gorged with blood. Sometimes considerable adhesion between, and thickening of the membranes, and minute and florid vessels upon the pia mater." 12.—Dr. Quin, after stating the vascular turgescence of the brains on dissection, goes on to say, that "in most of them a degree of inflammation had taken place, as appeared at the time of dissection, either by preternatural adhesions of the membranes, or by a partial opacity, and increased thickness of them, together with patches of inflammatory crust, very similar to those which are found on the abdominal viscera of persons whose death has been the consequence of *enteritis*, or on the lungs and pleura of those who have sunk under pulmonic inflammation."

From the modern pathological researches of M. Laennec, and several other continental physicians, it appears that, on minute examination of the brains of hydrocephalic patients, small tubercular granulations have been found dispersed through the cerebral and cerebellic mass, in the thalami nervorum opticorum, and even in the thickened portions of the meninges themselves. This interesting fact has been brought to light by the patient and laborious pathological investigations of our continental brethren, whose researches, *post mortem*, have been greatly facilitated by the peculiar economy of their public Institutions, and by the want of that morbid antipathy towards dissection, which cramps both public and private practitioners in this country. This morbid organization, discovered by glasses, and by minute anatomy, may explain much of the mystery which has hitherto hung over hereditary and local predisposition in hydrocephalic subjects, as also the connection between this disease and the scrofulous diathesis. The morbid organization described by Laennec and others, cannot justly be looked upon as the *effect*, so much

* Med. Chir. Journ. No. 3, p. 289.

as the *cause* of the hydrocephalic irritation, inflammation, and effusion.*

The most constant of all *post mortem* appearances is the effusion of water, sometimes into the ventricles, sometimes about the base of the skull, and sometimes between the dura and pia mater. This effusion is looked upon, by the best pathologists of the present day, as the *effect* of the stage of excitement, irritation, or inflammation; and as Nature appears to pour forth this fluid on the surface of various other tissues, for the present relief of a congested, inflamed, or irritated state of the parts, so it is considered, by men of great discernment, that the hydrocephalic effusion, though *ultimately* fatal in its consequences, gives a *temporary* relief to the general excitement, and the local inflammation. This idea is strongly supported by the mitigation of symptoms, commonly accompanying the effusion.

The fluid effused has this peculiarity, that it is not coagulable by heat, acids, or alcohol; a fact which was noticed by Fabricius Hildanus, and confirmed by Watson, Lecat, Mathey, Vieusseux, Haldat, and others. Haldat found, on evaporating the fluid effused in acute hydrocephalus, a brown residuum, consisting of muriate of soda, 96 ½ parts out of 100; the other 3 ½ parts consisted of water, albumen, mucus, gelatin, and phosphate of soda. It appears, therefore, that this fluid does not agree, in its chemical properties, with the fluids of other dropsies.

With the following observation on this subject, we were lately favoured by a distinguished physician of this metropolis, who has long directed his attention, with great success, to an investigation of the disease under review.

"The different effects produced by chemical tests in the water of hydrocephalus, and in the water of other dropsical effusions, I found verified in the *same individual.* The fluids were taken from an elderly gentleman, who had died, after long suffering, from diseased bladder and prostate. He had a hydrocele of one testicle; and water was also collected from the ventricles of the brain. The water from the *former* produced an immediate copious white curdy precipitate, by the addition of nitric acid. It was also rendered milky, and opake by heat; and when raised to the boiling point, it became thick, like jelly; while,

---

* See what Dr. Porter says on this subject, at page 291 of the third number of this Journal. See also a Dissection of Hydrocephalus, in the 4th vol. of the Medico-Chirurgical Journal, p. 427, where these scrofulous disorganizations were seen.

with the fluid from the ventricles of the brain, no alteration was produced by any degree of heat, nor by addition of nitric acid, whether made to it when it was cold or hot."

In the investigations, *post mortem*, of hydrocephalic patients, it has been too much the custom to limit the examination to the head, as the apparent, or at least, the supposed *fons et origo* of the disease. The spinal marrow, an important appendage to the brain, has been greatly neglected. Yet there is every reason to believe, from the symptoms during life, and the state of the medulla oblongata, on dissection, that the upper part of the spinal brain suffers in common with the inferior part of the cranial. In all Dr. Porter's dissections, the *tuber annulare* was involved in a mass of coagulable lymph, and some fluid escaped on dividing the medulla spinalis in its vertebral cell. It is not credible that the inflammatory action and its consequences should stop at a determined point on a continuous membrane; and the deranged functions of organs and parts supplied with nerves from the spine, afford presumptive proof that the spinal suffers in common with the cranial brain.

The abdominal lesions too, have been very much overlooked; for granting even that they were *consequential* of the cerebral affection, they are often destructive of life. Dr. Cheyne observes as follows:—"In the abdomen I have found the intestines inflamed, and *constricted from spasm,** and the surface of the liver of a bright red colour, abounding in minute vessels, and sometimes extensively adhering to the peritoneum. In several of the dissections I have found the surface of the liver studded with small white tubercles, not larger than a grain of mustard. The glands of the mesentery are often diseased." A little farther on Dr. Cheyne remarks, "upon dissection of hydrocephalic children, I have found in the liver the remains of great inflammatory action, and also proofs that undue irritation had existed in the alimentary canal." 12. *Essay I.*

Mr. Abernethy states his having examined three children who died of inflammation and effusion in the brain, "in all of which cases the liver was greatly diseased, and the bowels also exhibited a diseased appearance." "I have also, says this distinguished observer, examined a child who was supposed to have died of hydrocephalus, accompanied by a great disorder of the stomach and bowels.

* This constriction from spasm, indicates a pressure on the ori ins of the nerves supplying the constricted portions of intestine. Rev.

In this case, the bowels were inflamed, the liver sound, and the brain perfectly healthy in appearance." *Surgical Observations, Part Second.*

In nine dissections of hydrocephalus out of eleven, which Mr. A. T. Thompson made, there were evident marks of inflammation existing in the liver. In one of the remaining dissections, there was intus-susception of the jejunum; in the eleventh, inflammation of the colon. The same gentleman asserts, that in a majority of the cases of the disease, the organs of digestion are in fault, before the head appears in any degree affected. And he adds that, "in every opportunity which he had of observing the earliest approaches of hydrocephalus, the bowels have first become irregular; the stomach acescent, and the stools, whether procured by medicine or not, have been fœtid or clay-coloured, displaying defective action of the liver, and an imperfect formation of the bile."*

Dr. Boyton, many years ago, also, came to the conclusion, that hydrocephalus originated in the abdominal viscera, from finding that cathartics were the principal means of cure. *Cheyne's Second Essay, p. 50.*

Many instances might be cited, to prove that disease in almost any organ of the abdomen or thorax may give a developement to the hydrocephalic irritation. In the third volume of the Medico-Chirurgical Journal (Monthly Series) there is an interesting case of this disease, where, on dissection, the right *lung* was found studded with scrofulous tubercles, and six ounces of effusion in that side of the thorax, with marks of inflammation on the pleura. Here water was found in the ventricles of the brain, but no affection of the coverings.

In conclusion, after maturely and candidly examining the evidence and general sentiments of the profession, it appears that, in a great majority of instances, dissection has shewn *structural* derangements of other and distant parts accompanying hydrocephalus; and that in an equally great proportion of cases, the symptoms, during life, authorized the conclusion that *functional* derangements of these distant parts preceded the irritation, inflammation, and effusion in the head: — in other words, that hydrocephalus *symptomaticus* is infinitely more common than hydrocephalus *idiopathicus*.

Under these impressions, we conceive that the medical

---

* Edinburgh Medical and Surgical Journal, Vol. ii. p. 482.

profession is deeply indebted to Dr. Yeats for the steady and impressive manner in which he has kept the attention of medical practitioners directed to this most interesting point of pathology; and sincerely do we hope that this learned and enlightened physician will prosecute, with unremitted ardour, the track of investigation in which he has already distinguished himself.

### TREATMENT OF HYDROCEPHALUS.

*Theory* has always had a great influence on practice, and often produced incalculable mischief. Pathology is now the prime mover in therapeutics; but comparatively safe as is this guide, it may, and does occasionally, lead us astray. The circumstance of finding water effused in the ventricles of a hydrocephalic patient has led to very bad practice. Dr. Smyth, in a late work on this disease, appears to view the *effusion* as the great cause of the symptoms, and consequently that its *absorption* is the grand indication of cure. Here then is an instance where morbid anatomy has led to an erroneous practice. In ascending a link higher, and viewing the pathology of the disease as *inflammation*, we shall often go wrong; for something precedes even this. In short, unless we keep steadily in view the relation between the nervous and vascular systems—between the disorders of *function* in the early part of the disease, and the disorganizations of *structure* in the end, we shall never obtain a comprehensive and enlightened pathology, or a rational and successful practice, in this or in any other disease. The *methodus medendi,* in hydrocephalus, will always be vacillating too, while men pertinaciously adhere to confined notions, and partial views of the etiology of the complaint. We have endeavoured to show that hydrocephalus is not always, nor even generally, an idiopathic disease of the brain or its coverings; and that it does not arise *exclusively* from derangement of this or that organ, as some biassed practitioners believe, but *occasionally* from derangement of any one of them. The enlightened and unprejudiced observer, then, will examine, with all possible accuracy and minuteness, into the history of each individual case — into the previous and present state of each organ and function, and thereby endeavour to trace the morbid chain, link by link, from the first cause to the ultimate effect.

The great danger of viewing hydrocephalus as either *always* idiopathic or *always* symptomatic, consists in this, that the party so viewing it, though right in his opinion, will be exceedingly liable to err in his practice. Thus, suppose a man, who believes hydrocephalus to be an idiopathic affection of the brain, and the disordered functions of other organs at the time, merely symptomatic — and supposing that this is actually the case, which is the most favourable supposition, such practitioner will direct his remedies almost exclusively to the seat of the idiopathic affection, and not attach nearly sufficient importance to the sympathetic or consecutive derangements, though these last are, in fact, to be treated with *equal* care, as if they were the primary links of the chain. The same observation applies to the man who views hydrocephalus as symptomatic of abdominal disorder. He treats the cerebral affection as a secondary one, in point of consequence, though it deserves *equal* attention, as the visceral derangement at a distance. The golden rule, then, and that which is most easy of application, is, to direct the energy of our measures to the head or abdomen, in proportion to the urgency of the symptoms, in this or that region, always remembering, however, that in a great majority of cases, the functional derangement of the abdominal organs is the primary link in the morbid chain: and, that whether primary or secondary, its removal is the *sine qua non* of safety.

When we are called to a child labouring under functional disorder of the digestive organs, as evinced by unnatural secretions from the bowels, and some degree of morbid *irritability* in the whole system, but with no particular affection, apparently, of the sensorium, we should examine carefully into the history of the complaint—into the constitution, not only of the patient, but of the family—and into the state of the abdominal organs generally, by pressure on every point of the belly. If we find the abdomen tense, it is evident that the intestines are distended either with flatus or fecal accumulations; and this distention deranges the circulation of blood in the ventral viscera, besides keeping up a constant irritation in that region. This irritation is liable to be propagated to the brain, to which more blood is sent, in proportion to the difficulty of its transmission through the abdominal organs. The first indication here is self-evident. It is to clear the whole line of the intestines by such purgatives as may promote all the secretions; intestinal, pancreatic, and biliary. Two or three grains of calomel, followed in an hour or two by a solution of some neutral purging salt, should be administered, and repeated, if necessary, till the intestines are freely evacuated. The evacuations themselves will enable us to judge of the state of the bowels.

Their clayey, or variegated appearance, and offensive odour, will point out the necessity of *correcting* this morbid state of the secretions, as well as of evacuating them. But it frequently happens, that in every stage of hydrocephalus there is a most obstinate torpor of the intestinal canal, which torpor is, in all probability, owing to the state of the brain, and of the origins of the nerves supplying the abdominal viscera. This circumstance alone, even where the head is apparently unaffected, is a sufficient indication for leeching the temples, and applying stimulating frictions along the spine. By these means, and by the mercurial purgatives, we act both on the origins and extremities of the nerves in question, and thereby greatly promote the abdominal functions. But purgatives, local blood-letting, and spinal frictions, though they may, in many cases, succeed, are not to be exclusively trusted to. Continued purging by drastic medicines may even do harm by keeping up too much irritation in the digestive organs. In the intervals of purging, our aim should be to restore the morbid secretions to a more healthy condition, and this will be best effected by small doses of calomel, joined with antimonial powder, and where much irritability of the system prevails, even adding a small proportion of opium, in the form of the pulvis cretæ comp. c. opio. Hypothetical objections are formed against this medicine, under the idea that hydrocephalus was common inflammation of the brain. But it ought to be remembered that *irritation* in the system, and in that organ, often forms the very basis of the complaint, and, at all times, aggravates the inflammatory condition of the cerebral vessels. Half a grain of calomel, and a quarter of a grain of antimonial powder, with or without the opiate, ought to be given every three hours; and this small dose will be found to answer better than larger ones. Every day the bowels ought to be cleared by a saline or oily laxative, after the operation of which, the calomel should be again continued without interruption.

Where there is fullness or tenderness of the region of the liver, leeches should be applied to that region, in numbers proportioned to the degree of congestion there; and the tepid bath every evening will greatly assist the operation of the other remedies, especially if impregnated slightly with the nitro-muriatic acid.

All this is presupposing that the head has, as yet, evinced no particular symptoms of irritation or inflammation. The moment the cerebral disorder becomes manifest, or threatens in the least, then the most active means must be taken, in addition to those enumerated, to save the structure of the brain, and prevent effusion. These means are local or general blood-letting; cold to the scalp; blisters to the nape of the neck and spine; frictions; elevation of the head; quietude; darkness.

The jugular vein or temporal artery may be opened; but in general, leeches, in sufficient number, not less than from eight to twelve, should be applied to the temples, nape of the neck, and upper portion of the spine; immediately after which, a long and narrow blister should be applied from the occipital foramen to the upper dorsal vertebra, and a discharge kept up by savine cerate or unguentum lyttæ. The head must be shaved, and clothes wet with vinegar, water, and a little spirit, should keep up a constant abstraction of heat from the scalp. This process is never to be neglected, though it too often is so, while the stage of irritation or excitement lasts.

During this time, the mercurial plan, to correct the secretions and keep the intestinal canal clear, is not to be interrupted; and as the liver is very frequently in fault, *mercurial liniment* should be well rubbed along the spine and region of the liver, every time the child comes out of the tepid bath, which should not come higher than the pit of the stomach. The mercurial liniment is one of the best stimulants that can be applied to the spine, as it answers a twofold purpose, that of exciting the energy of the nerves arising from the medulla spinalis; and of entering the system, so as to act upon the hepatic and other secretions, and thereby relieve the head.

"I have myself, says Dr. Cook, in his learned researches on this subject, witnessed the good effects of mercury used both externally and internally, in several cases of what I supposed to be water in the brain; of these, two were marked by the symptoms of confirmed hydrocephalus. On the whole, the general opinion appears to be in favour of the use of mercury. Indeed, Dr. Abercrombie seems only to deny its specific influence, and to object to the indiscriminate employment of it. The instances of recovery from this dangerous complaint, under the management of Dobson, Perceval, and many others, as well as those which have fallen under my observations, may, I think, be fairly adduced in support of the practice in question; and I believe few physicians of the present day would think themselves justified in omitting to employ mercury in attempting the cure of this complaint." 466.

The effects of digitalis in the stage of excitement are very doubtful, and after effusion has taken place, still more so. It is a medicine, however, which is pretty generally employed, on the principle of arresting the velocity of the circulation, by checking the inordinate action of the heart. Dr. Cheyne informs us, that he begins with a dose of eight or ten drops of the saturated tincture, every six hours, increasing it gradually by two or three drops at a time, till constitutional symptoms supervene.

It ought ever to be borne in mind, that the symptoms of actual *effusion*, in hydrocephalus, are sometimes very equivocal; and may be closely imitated by turgescence of the cerebral vessels. This, coupled with the almost certain fatality of hydrocephalic effusion, should induce us not to desist from the depleting plan, so soon as the symptoms of effusion manifest themselves. It is moreover ascertained by dissection, that the inflammatory condition of the cerebral vessels does not always, nor even generally, subside when effusion takes place. We have opened many heads, where water was effused, and yet inflammation was going forward. Nor is this peculiar to the head; the same happens in the chest and other parts. We this day [16th of April, 1819,] opened a body, where the immediate cause of death was effusion in the lungs, cavity of the pleura, and into the pericardium; yet the internal surface of the pericardium, and the exterior surface of the heart were in the highest possible state of inflammation, notwithstanding the effusion of serum and coagulable lymph in all directions.

It is evident that in hydrocephalus, as well as in any other disease, where effusion is going on from irritation, turgescence, or inflammation, if we arrest the depleting plan, at such a juncture, we only give increased power to the disease.

Whether, when effusion is complete, and the inflammation reduced, the effused fluid is ever absorbed from the ventricles of the brain, is exceedingly problematical. We should suppose that slight effusions may be absorbed, especially from between the coverings of the brain. Because there are few or no absorbent vessels traceable in the cerebral mass, it does not follow that no absorption can take place, since recent experiments prove that the VEINS can perform that office.

The Vapour bath has lately been recommended by some continental physicians; and a few cases are published, where this measure is said to have proved successful, even where effusion had actually taken place. A case is related in the Medical Commentaries for 1782, where the vapour bath, as is attested by Doctor William Hunter, restored a child, after all the symptoms of effusion had appeared in the worst degree.

We are disposed to hope, that in this short *Exposé* of the therapeutical means to be employed in hydrocephalus acutus, will be found most of those that are really useful, and easily applicable to practice. We have been more anxious to investigate the nature and pathology of the disease, than to enumerate a long list of remedies, or to lay down a systematic code of treatment. The latter must vary in almost every individual case; and the man who entertains the most comprehensive views of the pathology, will always be most capable of adapting the remedial means to the end or objects desired.

It will also have been observed, that the foregoing Paper has not been drawn from any particular source — nullius addictus jurare in verbo magistri — but that we have ranged freely over the field of medical literature, while no inconsiderable share of it is the result of personal observation and reflection. Yet we cannot conclude, without taking some exclusive notice of Dr. Cheyne's work, which did not come to hand till the greater part of this article had been prepared for the press, although many of his statements are quoted from the first edition of his Essays. The present republication contains a great mass of important observations and highly interestiug cases, (some of them new) which eminently entitle it to the study of the Profession. From the Second Essay of this very able and experienced Author, we shall introduce to our Readers a few Extracts relative to the treatment of the disease under consideration.

" The state of the hypochondria, the nature of the stools, and the other excretions, the appearances of the tongue, and the smell of the breath, ought to be examined with care. If the patient wince when the right hypochondrium is pressed, leeches ought to be applied to it, or the margin of the ribs may be cupped and scarified; if there be much pyrexia with head-ach, blood must be drawn from a vein, or from the temporal artery. Then cathartics are to be given, to promote, and, if necessary, to alter the secretions; generally calomel, with small doses of some common purgative of an active kind, as rhubarb, jalap, or scammony, with aloes. If there be a sickly smell of the breath, and fullness and uneasiness at the pit of the stomach, an irritation of the mucous membrane of the intestines is denoted, which is sometimes relieved by mild antimonials; these consequently are to be added to the cathartics; squill may also be exhibited with the same view, more especially when the urine is deficient. If the stools be dark green and glairy, most probably the common cathartics will have little effect; indeed, we cannot expect that they will change the nature or appearance of the secretion, which issues from an organ over which they have little controul.

"Even calomel, the medicine from which most might be expected, is sometimes inert as a purgative, and has no influence over the system as a mercurial; and this seems to arise from want of suitable preparation. In the cases in which hydrocephalus seems most remarkably to have its source in a disorder of the abdominal viscera, and in which the cure is to be effected by exciting these organs to free secretion, we are generally unable, after the first day or two, to effect that purpose by direct means.

"It is a well known law in pathology, that if a gland be excited beyond a certain point, it is no longer able to perform its secerning function; and when so circumstanced, a stimulus applied to it, instead of restoring secretion, often increases the vascular excitement upon which its interruption depends. In hydrocephalus, the biliary secretion is generally languid as well as vitiated; and the presumption is strong, that this condition of the bile depends upon the general vascular excitement of the liver. If the viscera of the abdomen, and particularly the liver, are in a high state of irritation, this irritation ought to be allayed before the stimulus which increases their secretions can be employed with advantage, or even with safety. The true practice is, in the first place, to reduce arterial action by venesection, or by topical bleeding and blistering, and then to restore the secerning function of the viscera by means of calomel and other cathartics.

"In high degrees of vascular excitement of the liver, as, for instance, in hepatitis, mercury, which is the specific remedy, is beneficial or injurious, according to the condition of the liver when it is administered. It is injurious till, as Dr. Johnson says, "the inflammatory congestion in the liver is relieved by free blood-letting."* I formerly quoted a passage of a similar import from Dr. Macgregor's Medical Sketches. The power of blood-letting in forwarding the operation of mercury, might be shewn by a number of cases and observations. In the endemic hepatitis, described by Mr. Chisholme,† the practice pursued, with remarkable benefit, was to let blood freely in the first instance. "After the third bleeding," he remarks, "we gave from two to seven grains of calomel, with from one-fourth of a grain to a whole grain of opium, three times a day. This practice, continued for two days, brought on a copious salivation; when this was effected, we considered the patient out of all danger, and it was astonishing how readily cases of the most dangerous tendency were cured by this method in a few days."

When hydrocephalus appears to be a secondary disease, our Author recommends us to begin the cure by attempt-

* Influence of Tropical Climates, &c. p. 282.

1s t Volume, 2d Decade, Duncan's Medical Commentaries.

ing "to relieve the irritation of the abdominal viscera, which is never fully accomplished until their healthy secretions are restored by cathartics." "The cathartics applicable to hydrocephalus are those which increase the secretions. We may remove fæces from the alimentary canal, by quickening the peristaltic movements, with but little benefit to the patient. Our object is to change the action of the secerning vessels, and more especially encourage and improve the biliary secretion; but in so doing we must avoid irritating the mucous membrane of the intestines."

As the milder antimonials have the effect of increasing the secretions of the liver and primæ viæ, Dr. Cheyne recommends them in combination with mercurials.

Our Author has added a series of cases, in which, after depletion, opium, in conjunction with mercurials and antimonials, was given with the best effects. He observes, that in diseases attended with increased activity of the circulation, a very moderate dose of opium will be found sufficient. The same is applicable to affections of the bowels, where opium, in excess, might interfere too much with their peristaltic movements. "If it be wished (says Dr. Cheyne) to place the constitution suddenly under the influence of mercury, I know but of two means, apparently opposite, namely, blood-letting and opium." This we can confirm from pretty extensive observation.

Dr. Cheyne introduces some euthanasial hints relative to the effects of blood-letting in mitigating sufferings, where we are without a prospect of cure. He has long been convinced that patients who die of organic diseases of the brain, struggle and suffer much more when they are not bled than they would do, were venesection performed. It is well known, indeed, to our West India practitioners, that blood-letting smoothes the way to the grave, without quickening the pace of the victim of yellow fever. "The convulsions (says Dr. Cheyne) which attend hydrocephalus may be removed by affusion, or rather aspersion of the face and neck with cold water;" which he thinks is preferable to the warm bath, which is more operose, and less effectual. He recommends "the injection of a large glyster, made by dissolving phosphate of soda or sulphate of magnesia in broth, after which children in hydrocephalus generally lie easy for a couple of hours."

We are sorry that the great length to which this article has run, prevents our introducing any of the great mass of highly valuable cases appended to this volume; but we are in hopes that most practitioners will peruse the work itself, which is in a compendious form and small type, worthy of imitation in the present period of general distress.

To Dr. Yeats's Work and Appendix, together with Dr. Cooke's erudite Compilation, we would also wish to draw the attention of our brethren; and if they derive from their perusal as much pleasure and information as we have done, they will be amply repaid for their labour.

## Figures and Tables

**Figure f1:**